# Visualisation of DCP, a nerve agent mimic, in Catfish brain by a simple chemosensor

**DOI:** 10.1038/s41598-018-21780-5

**Published:** 2018-02-21

**Authors:** Himadri Sekhar Sarkar, Ayndrila Ghosh, Sujoy Das, Pulak Kumar Maiti, Sudipta Maitra, Sukhendu Mandal, Prithidipa Sahoo

**Affiliations:** 1 Department of Chemistry, Visva-Bharati University, Santiniketan, 731235 India; 2 Department of Microbiology, University of Calcutta, Kolkata, 700019 India; 3 Department of Zoology, Visva-Bharati University, Santiniketan, 731235 India

## Abstract

A chemosensor, 3-aminophenol-based rhodamine conjugate (ARC) has been developed for visualisation of diethylchlorophosphate (DCP), mimic of a chemical warfare agent, in Catfish brain. The simple detection of DCP by “turn-on” fluorescence property of the chemosensor makes it unique for easy and rapid *in vivo* and *in vitro* detection of DCP with the detection limit of 5.6 nM.

## Introduction

1995, The terrorist attack on Tokyo subway introduces the whole world with a new threat to mankind- Chemical Warfare Agents (CWAs)^1–3^. The simple organophosphates, present in pesticides eventually become more popular chemical weapon due to its very simple method of manufacturing, availability, and low cost along with its dispensability. Despite of its common use as a pesticide, various analogues of such organophosphates are found to be very potent nerve agent, which irreversibly damage functions of nerve cells. Among all the highly toxic, volatile nerve agents, Sarin (GB), Soman (GD) and Tabun (GA) are of most common. If the nerve agents are being inhaled or absorbed through skin, their reactive phosphate group irreversibly react with the hydroxyl group of cellular acetylcholinesterase, which is responsible for breaking down the acetylcholine neurotransmitter, leads to its inactivation. The fatal consequences are neurological imbalance at cholinergic synapses, failure of several organs, paralysis of central nervous system and rapid death^4,5^. On 4^th^ April 2017, Khan Shaykhun, a city of Syria faced the destructive activities of suspected chemical warfare with nerve agent Sarin^6^. For public safety concern and national security, a reliable, facile and reactive detection method of such nerve agents is of great global interest towards the development of practical ‘in field’ devices.

Till today, several detection methods, such as- colorimetry^7–10^, fluorometry^11–17^, interferometry^18^, electrochemistry^19–22^, surface acoustic wave (SAW) devices^23,24^, photoacoustic spectroscopy^25^, moleculer imprinting coupled with lanthanide luminescence and fibre optics^26–28^, enzymatic assay^29,30^, and gas chromatography-mass spectrometry^31^ have been explored to detect substantial number of CWAs. But the problem arises when these existing methods show one or more drawbacks such as low response and sensitivity, limited selectivity, non-specificity and non-portability, difficulties in real time detection, false positive readings and operational complexity. The chemosensors mediated detection is an extremely convenient and simple method based on optical outputs such as color change or change in fluorescence intensity after the exposure to the target analytes^32–35^. Chemosensors make such detection method attractive and widely acceptable by fulfilling the key features of a practical sensing system i.e. high selectivity, sensitivity, specificity, portability, ability of real time sensing along with cost effectiveness^36,37^. Implementation of a reliable chemosensor is a highly challenging but it brings a considerable importance for their wide application in medical, environmental and biological field. Generally, diethylchlorophosphate (**DCP**) is used as mimic of the nerve agent Sarin. **DCP** is much less toxic than Sarin but its mode of action is similar.

With this stand point in this particular research field, we have described a chemosensor **ARC**, 3-aminophenol based rhodamine conjugate. This chemosensor is simple and efficient in showing fluorescence signal in presence of **DCP** in aqueous medium both in *in vivo* and *in vitro* systems. There are reports that described recognition of deadly and relevant molecules like **DCP** and other organophosphates. A comparative assessment of our chemosensor has been provided with that of the published one (Table S1). In our present work with the help of a very simply designed chemosensor we established the detection of **DCP** in a direct, fast and selective way in living system. Over usage of organophosphate pesticides or use of similar compounds through chemical warfare can essentially disseminate such compound in ecosystem. Hence detection of such compound from contaminated samples is very much awaiting. With a unique approach, we have applied the probe **ARC** in catfish brain to visualize **DCP** which was also treated subsequently and the success of this experiment help to advance its practical field-application. As per our knowledge this is the first report where a chemosensor is used for visualizing **DCP** *in vivo*.

## Results and Discussion

### Design and scheme

**Figure 1:**
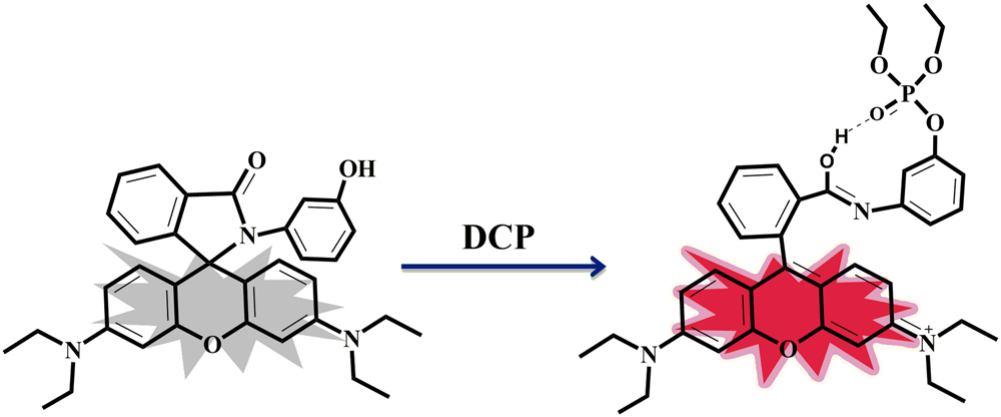
“Turn-on” fluorescence sensing mechanism of **ARC** upon addition of **DCP**.

Probe **ARC** was synthesized from a simple reaction of rhodamine B and POCl_3_, followed by reaction with 3-amino phenol with 74% overall yield (detailed in method section). Characterization of the probe **ARC** has been done by ^1^H NMR, ^13^C NMR, ^31^P NMR and MS analysis (Figs S1–S3). **ARC** shows fluorescence “turn-on” response upon binding with **DCP** in aqueous medium at neutral pH (Fig. 1). Several studies such as absorption, fluorescence, NMR titrations and TDDFT calculations have been carried out to establish the interactive properties of **ARC** towards **DCP**, our target analyte.

### UV-vis and fluorescence spectral studies

**Figure 2:**
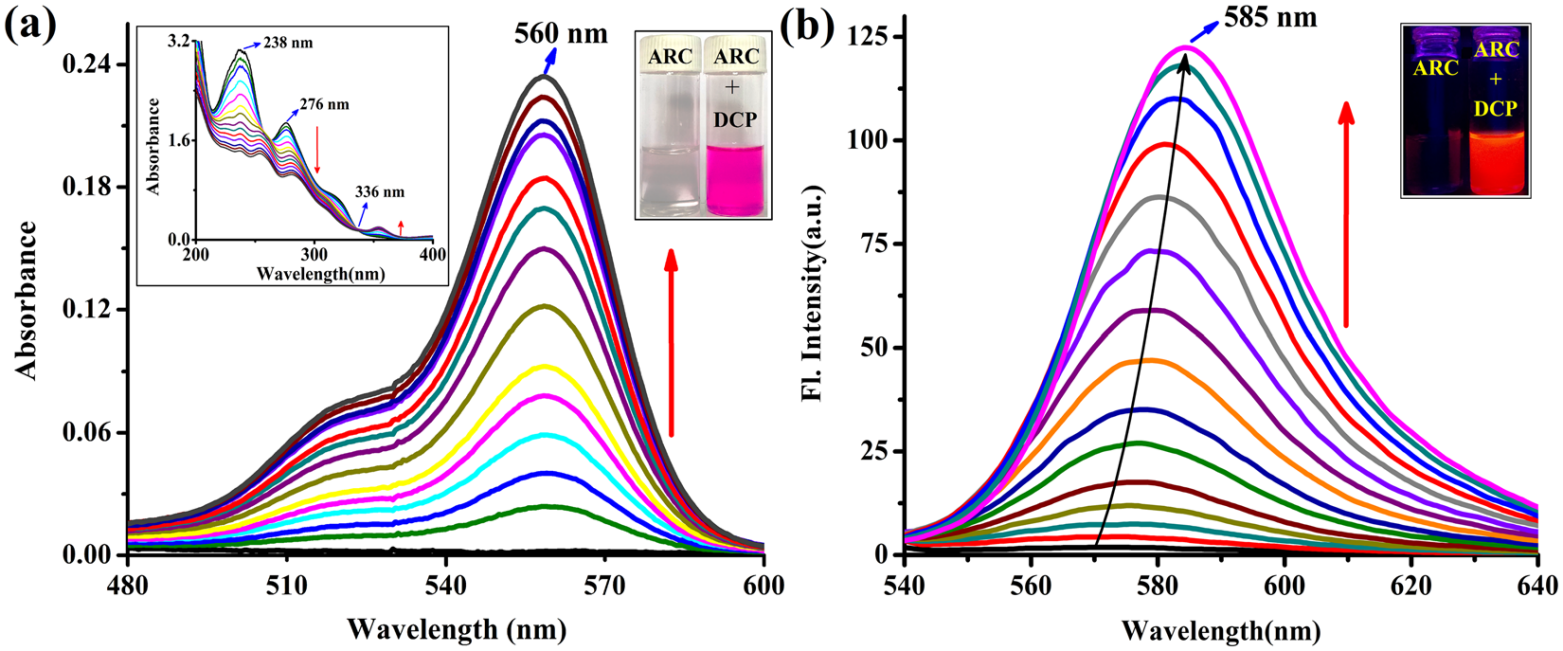
(**a**) UV-vis absorption spectra of **ARC** (1 µM) upon gradual addition of **DCP** upto 1.1 equiv. in H_2_O-CH_3_CN (10:1, v/v). [Inset: left- UV-vis spectra of the region 200–400 nm, right- photograph of visual color changes of **ARC** and **ARC** + **DCP** complex in aqueous medium] (**b**) Fluorescence emission spectra (λ_ex_ = 520 nm) of **ARC** (1 µM) toward **DCP** at varied concentrations (0, 0.01, 0.05, 0.10, 0.15, 0.20, 0.30, 0.40, 0.50, 0.60, 0.70, 0.80, 0.90, 1.0, 1.05, 1.10 µM) in H_2_O-CH_3_CN (10:1, v/v). [Inset: Fluorescence color changes of **ARC** and **ARC** + **DCP** complex in aqueous medium].

From the UV-vis absorption spectra of the chemosensor **ARC** it has been revealed that the addition of **DCP** gradually enhanced the absorption of **ARC** in aqueous medium at neutral pH. The UV-vis absorption spectra of the probe **ARC** showed a prominent isosbestic point at 336 nm that proves stronger complexation with three absorption maxima at 238, 276, 355 nm through change in color from colorless to deep pink along with gradual enhancement of **ARC** absorption during **DCP** accumulation (Fig. 2a).

**ARC** shows a very weak fluorescence quantum yield (Ф_fs_ = 0.006). Within a few seconds in contact with **DCP**, **ARC** gives a prompt fluorescence response with a full signal emission maxima at 585 nm (λ_ex_ = 520 nm) - a 100 fold increased fluorescence signal (Ф_fs_ = 0.58) compared to its own (Fig. 2b, Table S2). Non-linear fitting analysis from fluorometric titration curves deduced the binding constant of **ARC**-**DCP** complex is 7.89 × 10^6^ M^−1^ (Fig. S4). This fluorometric titration of **ARC** with **DCP** also results the detection limit of **ARC** which is 5.6 nM of **DCP** (Fig. S5). All these results explained the deep pink color development of the **ARC** from colorless and non-fluorescent entity, through the ring opening process of the spirolactam ring of the probe by irreversible coordination with **DCP**.

Selectivity test has also been performed with different organophosphates (OPs) e.g. diethylcyanophosphonate (DCNP), diethyl(1-phenylethyl)phosphonate (DPEP), diethyl(methylthiomethyl)phosphonate (DMTMP), diethyl-(2-oxopropyl)phosphonate (DOPP), Ethyl methylphosphonate (EMP), Diethyl methylphosphonate (DEMP) by UV-vis and fluorescence method (Figs S6 and S7). Interestingly probe **ARC** can only recognise **DCP** distinctly among all of the OPs (Fig. S8). We have also investigated the fluorescence behaviour of **ARC** on the addition of various interfering substances like dimethylmethylphosphate (DMMP) - a nonreactive analogue of **DCP**; some common metal ions such as Zn^2+^, Cu^2+^, Co^2+^, Hg^2+^; reactive oxygen species (ROS), i.e. NaOCl, ^t^BuOOH, H_2_O_2_; common acid chlorides, i.e. acetyl chloride, benzoyl chloride; and common pesticides, i.e. benzene hexachloride (BHC), phorate and chlorothalonil (Fig. S9). From the comparative fluorescence experiment we can conclude that there is no interference of the above mentioned substances with **ARC**. So, it’s clear that **ARC** can be a simple, selective and efficient chemosensor which can directly measure **DCP** by a fluorescence “turn-on” mechanism.

A kinetic investigation of the reaction between **ARC** and **DCP** showed that the reaction is complete within 20 seconds (Fig. S10). We then examined the kinetic profiles of the reaction under pseudo-first-order conditions with a large excess of **DCP** (10 equiv.) over the chemosensor **ARC** (1 µM) in aqueous medium (at neutral pH) at room temperature. The pseudo-first-order rate constant (*k′*) has been calculated as *k′ = *0.172 s^−1^ (Fig. S11). This rate constant (*k′*) has been calculated according to the equation^38^:

$$\mathrm{ln}[({{\rm{F}}}_{{\rm{\max }}}-{{\rm{F}}}_{{\rm{t}}})/{{\rm{F}}}_{{\rm{\max }}}]=-k^{\prime} t$$

where F_t_ and F_max_ are the fluorescence intensities at 585 nm (λ_ex_ = 520 nm) at time ‘t’ and the maximum value obtained after the reaction is complete, respectively, and *k′* is the observed pseudo-first order rate constant.

### Vapour phase detection studies

Sensing ability of **ARC** to **DCP** has also been successfully established in gaseous phase, as our probe can satisfy an important role of a convenient chemosensor kit. We did the experiment using a Whatman-41 filter paper strip immersed in **ARC**, keeping inside in a glass chamber. A rapid “turn-on” fluorescence response was noticed after adding a few drops of **DCP** in that glass chamber (Fig. S12). From this observation it has been concluded that **ARC** strongly binds with **DCP** even from gaseous phase.

### pH titration studies

As rhodamine derivatives are very sensitive in acidic medium, we are always aware to maintain pH while doing experiments with rhodamine derivatives. In acidic condition the spirolactam ring opens up to show its original deep pink color. So it’s very important to fix the pH range where rhodamine derivatives will be non-fluorescent. From the pH titration it has been shown that **ARC** is stable and non-fluorescent at pH range 5–13. It means the spirolactam ring of **ARC** opens in highly acidic condition (below pH 5). We therefore maintained the pH of **ARC** at 7 while doing all experiments. However, while adding **DCP** at neutral pH the probe shows its deep pink color even at pH 8 (Fig. S13)^39,40^, which demonstrates the practical applicability of the chemosensor.

### NMR titration studies

Interactive properties of the chemosensor **ARC** towards **DCP** was further investigated via ^1^H, ^13^C, ^31^P NMR titrations carried out using solvent DMSO-d_6_. Upon **DCP** addition, aromatic protons of the xanthene moiety of **ARC** display downfield shift. This is due to the decrease in electron density of the xanthene moiety upon binding with **DCP** (Fig. S14). In ^13^C NMR titration the aromatic region of **ARC** becomes downfield while comparing with the original one (Fig. S15). The spiro cycle carbon peak at 67 ppm was shifted to 143 ppm. This coordination led to the spiro cycle opening and changes to the absorption and emission spectra. From ^31^P titration it has also been shown that the only one singlet peak of **DCP** at −0.39 ppm drastically shifts to lower frequency −5.06 ppm upon binding with **DCP** which indicates that there is a strong interaction between **ARC** and phosphate group of **DCP** (Fig. S16).

### Density Functional Theoretical (DFT) studies

**Figure 3:**
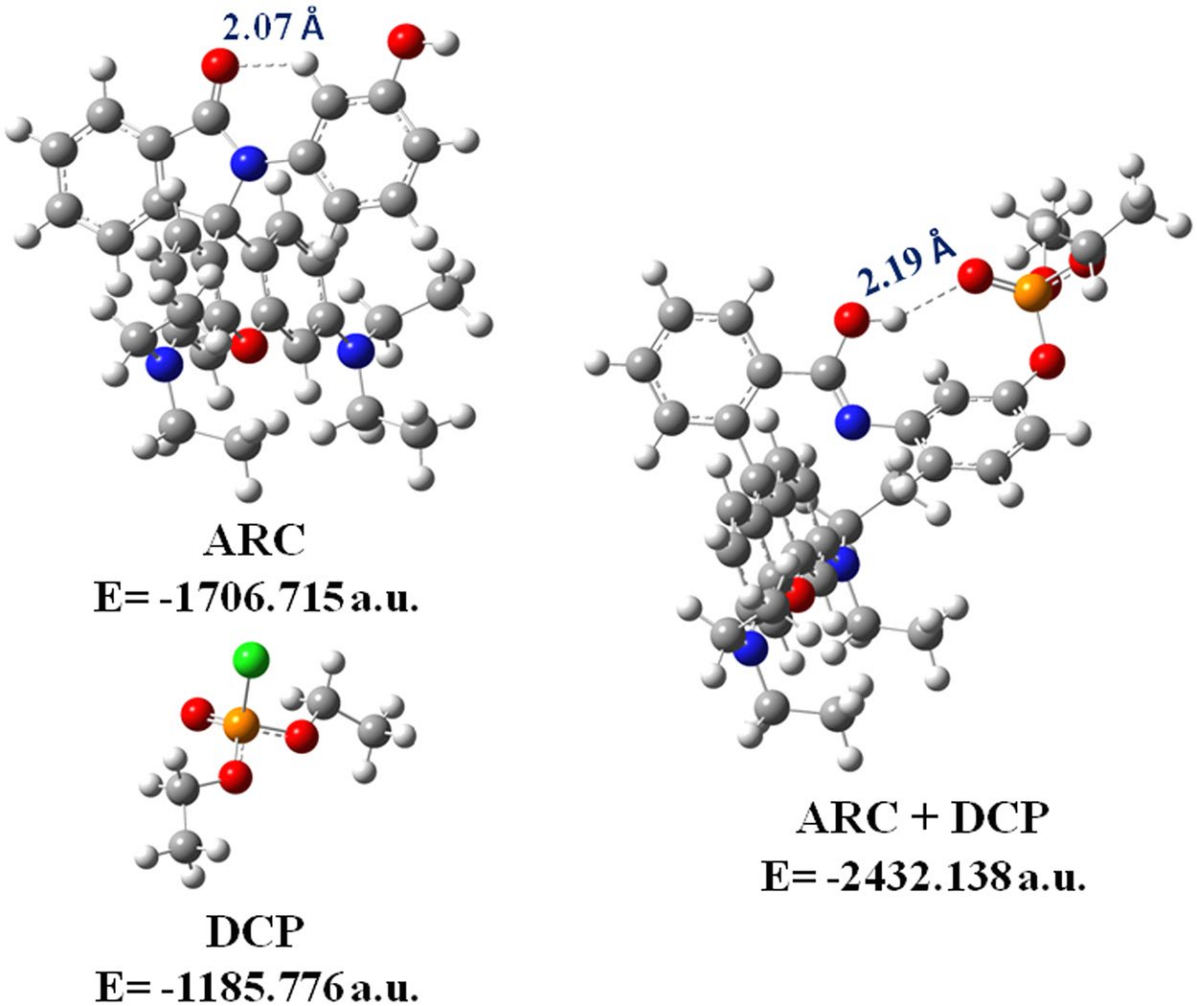
Optimized structures of **ARC**, **DCP** and **ARC-DCP** complex with their corresponding energies. The hydrogen bond interactions are denoted by dotted lines.

The interaction of the receptor **ARC** with **DCP** has further been comprehended by quantum chemical calculations at the TDDFT level with the B3LYP/6-31 + G(d,p) method using the Gaussian 09 program (Tables S3 and S4). The optimized structures and their corresponding energies are shown in Fig. 3. The energy gap between the HOMO and LUMO of the **ARC**-**DCP** complex structure is smaller (3.01 eV) than that of the chemosensor **ARC** (4.61 eV) (Fig. S17). The main contributing transitions for S_0_ → S_1_ energy state arises from HOMO-1 → LUMO. This is consistent with the absorbance band at 560 nm obtained experimentally.

### Imaging of Catfish brain and A549 cells

**Figure 4:**
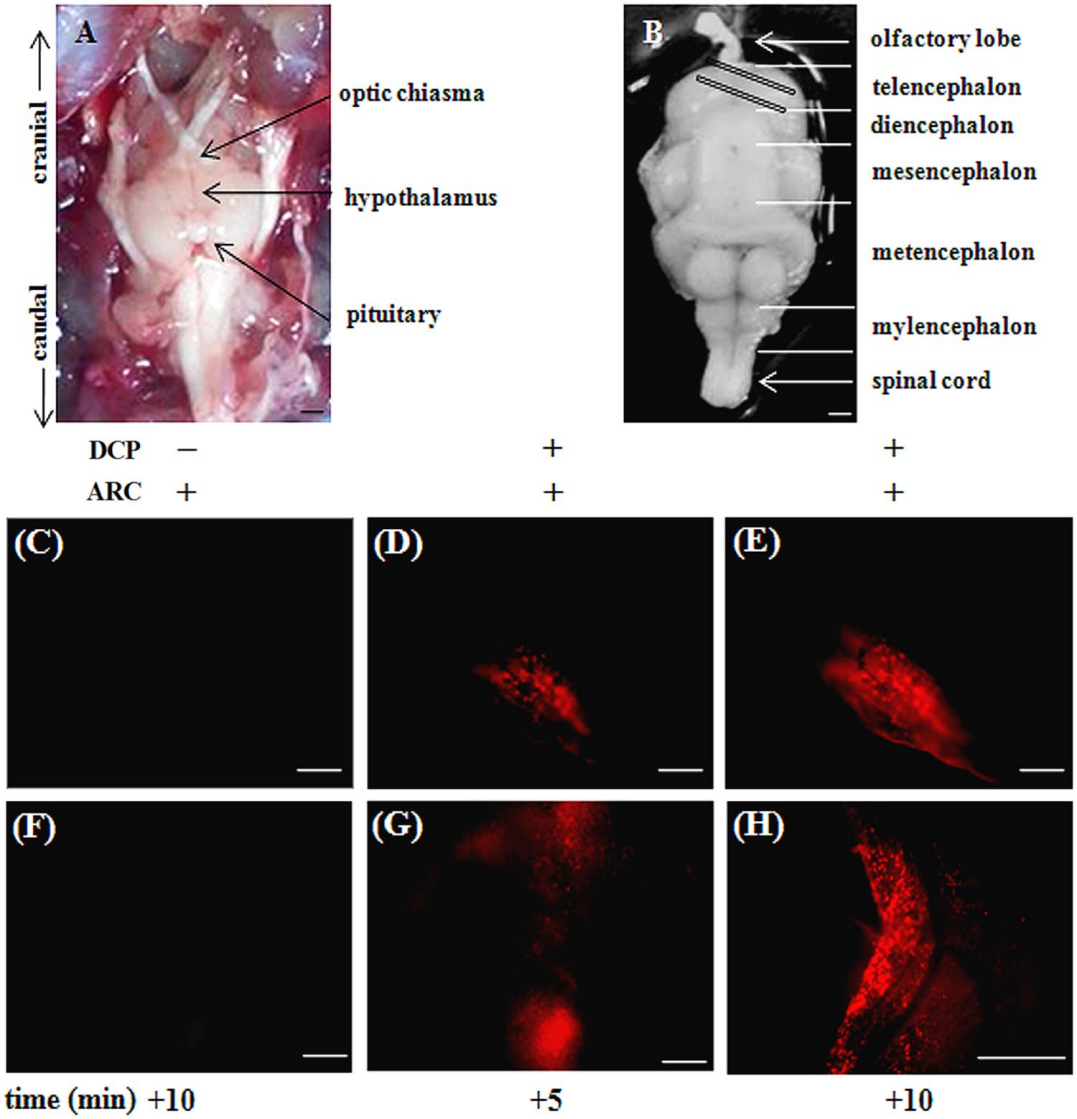
Representative photomicrographs of Catfish brain (ventral side up) showing cranial and caudal ends, hypothalamus, pituitary and optic chiasma during dissection (**A**) and after harvesting in chilled PBS (**B**). Brain slices from telencephalon (marked as diagonal rectangles in (**B**) were pre-incubated with **ARC**, washed with PBS, treated with the analyte **DCP**, washed again to remove excess **DCP** and observed under fluorescence inverted microscope at 5 min intervals (**D**,**E** from upper slice and **G**,**H** from lower slice). Parallel slices treated with **ARC** only served as negative control (**C**,**F**). Figures are representative of three independent observations from separate donors, scale bar ~1 mm (**A**,**B**); ~200 µm from (**C** to **G**) and ~400 µm for (**H**).

Furthermore, we attempted to visualize **DCP** from Catfish brain through the fluorescence microscopic imaging upon binding with **ARC** in Catfish brain. To test the efficiency of binding of **DCP** to **ARC** and subsequent fluorescence emission, fish brain, more specifically the slices from optic tectum– the region which have earlier been shown to possess high level of acetylcholinesterase activity^41^, were used in the present study. As shown in Fig. 4, incubation of the brain slices with the chemosensor **ARC** alone does not produce detectable fluorescence signal, while incubation of **ARC** followed by **DCP**, promoted a robust increase in fluorescence emission indicating specificity of interaction. The experiment has also been done in reverse way, i.e. first incubation of **DCP** followed by **ARC** which gives the same result as earlier. These also suggest successful penetration of **ARC** and **DCP** into the Catfish brain tissue.

In addition, we have also tested permeability and toxicity of **ARC** in A549 human cancer cell line (Fig. S18). It is also revealed successful entrance and detection of **DCP** by the probe **ARC**. The **ARC** doesn’t show major cytotoxicity (Fig. S19)^42^ to the tested cells and could be a choice of probe to detect **DCP** in simple and complex cellular matrix.

## Conclusion

In summary, we have successfully developed a rhodamine based chemosensor **ARC** for *in vivo* and *in vitro* detection of **DCP** with very low concentration at neutral pH. The simple detection method exhibits high sensitivity of **ARC** towards **DCP** both in solution and gas phase and the interaction of **ARC** with **DCP** can even noticed with nacked eye. The above method was successfully applied to visualize **DCP** in catfish brain through “turn-on” fluorescence signal. This proves the potentiality of the probe **ARC** as a simple, fast, cost-effective and very useful tool to determine **DCP** in biological samples.

## Materials and Methods

### Materials

Rhodamine B, phosphorus oxychloride and 3-amino phenol were purchased from Sigma-Aldrich Pvt. Ltd. (India). Unless mentioned otherwise, materials were obtained from commercial suppliers and were used without further purification. Solvents were dried according to the standard procedures. Elix Millipore water was used throughout all experiments. ^1^H and ^13^C NMR spectra were recorded on a Bruker 400 MHz instrument. For NMR spectra, DMSO-d_6_ and for NMR titration DMSO-d_6_ and D_2_O were used as solvent using TMS as an internal standard. Chemical shifts are expressed in δ ppm units and ^1^H–^1^H and ^1^H–C coupling constants in Hz. The mass spectrum (HRMS) was carried out using a micromass Q-TOF Micro^TM^ instrument by using methanol as a solvent. Fluorescence spectra were recorded on a Perkin Elmer Model LS 55 spectrophotometer. UV spectra were recorded on a SHIMADZU UV-3101PC spectrophotometer. The following abbreviations are used to describe spin multiplicities in ^1^H NMR spectra: s = singlet; d = doublet; t = triplet; m = multiplet.

### Preparation of ARC

**Figure 5:**
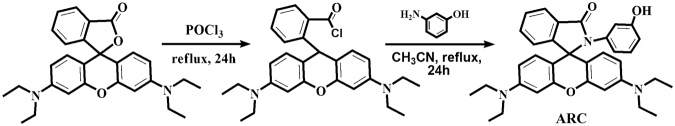
Synthesis of the chemosensor **ARC**.

Rhodamine B (4.80 g, 10 mmol) was taken in a 250 mL RB flask containing 100 mL freshly distilled POCl_3_ and allowed to reflux for 24 h under a dinitrogen blanket. The excess amount of POCl_3_ was removed in a rotary evaporator. The acid chloride was dried under vacuum and used for the next step without further purification. This was dissolved in dry acetonitrile (100 mL) then 3-amino phenol (1.63 g, 15 mmol) followed by triethylamine (5 mL) were added slowly. Once the addition was complete, the resulting mixture was heated to reflux for 24 h. The reaction mixture was then concentrated in a rotary evaporator under low pressure and then extracted with dichloromethane. After drying it over anhydrous Na_2_SO_4_, the organic layer was evaporated completely. The residue was purified by column chromatography with the eluent chloroform: ethyl acetate (6:1, v/v) to get the product **ARC** with 74% yield (Fig. 5). **ARC** was then recrystallized from methanol as a pale pink solid. ^1^H NMR (400 MHz, DMSO-d_6_): d (ppm) = 9.34 (-OH, s, 1 H), 7.84–7.86 (d, 1 H, J = 8 Hz), 7.51–7.55 (m, 2 H, J = 16 Hz), 7.00–7.02 (d, 1 H, J = 8 Hz), 6.88–6.92 (t, 1 H, J = 16 Hz), 6.47–6.52 (m, 3 H, J = 20 Hz), 6.35–6.40 (m, 3 H, J = 20 Hz), 6.280–6.286 (d, 2 H, J = 2 Hz), 6.19–6.20 (d, 1 H, J = 4 Hz). ^13^C NMR (400 MHz, DMSO-D_6_): d (ppm) = 167.18, 157.54, 154.15, 152.65, 148.76, 138.42, 133.74, 129.95, 129.29, 128.82, 124.16, 123.26, 116.78, 114.14, 113.75, 108.55, 106.18, 97.72, 44.07, 12.87. HRMS (TOF MS): (m/z, %): Calcd. for C_34_H_35_N_3_O_3_: 533.27. Found: m/z = 534.27 (M + H^+^; 100%). ^1^H, ^13^C NMR and HRMS spectra are shown in Figs S1–S3.

### UV-vis and fluorescence titration

A stock solution of **ARC** (1 × 10^–6^ M) was prepared in water-acetonitrile (10:1, v/v). 1 × 10^−5^ M **DCP** solution was prepared in Millipore water. All experiments were carried out in aqueous medium at neutral pH. During titration, each time a 1 × 10^−6^ M solution of **ARC** was filled each time in a quartz optical cell with 1 cm optical path length and **DCP** stock solution was added into the quartz optical cell gradually by using a micropipette. For all fluorescence measurements, excitations were provided at 520 nm, and emissions were collected from 540 to 640 nm.

### Kinetic Study

The kinetic study of the reaction between **ARC** and **DCP** has been done by measuring the fluorescence spectra after mixing **ARC** and **DCP** in a cubic 4-sided quartz cell of 3 ml. The reaction was carried out at room temperature under the excess amount of **DCP** (initial concentration [**ARC**] ≪ [**DCP**]) and the reaction was expected to reach 100% conversion of chemosensor **ARC** to **ARC**-**DCP** complex. **ARC** and **DCP** solution of concentration 1 µM and 10 µM, respectively, were prepared in aqueous medium and mixed to investigate the kinetic. The excitation wavelength was 520 nm.

### Live Cell Imaging

#### Cell Culture

A549 cell (Human cell A549, ATCC No CCL-185) lines were prepared from continuous culture in Dulbecco’s Modified Eagle’s Medium (DMEM, Sigma Chemical Co., St. Louis, MO) supplemented with 10% fetal bovine serum (Invitrogen), penicillin (100 μg/mL), and streptomycin (100 μg/mL). Cells were initially propagated in 75 cm^2^ polystyrene, filter-capped tissue culture flask in an atmosphere of 5% CO_2_ and 95% air at 37 °C in CO_2_ incubator. When the cells reached the logarithmic phase, the cell density was adjusted to 1.0 × 10^5^ per/well in culture media. The cells were then used to inoculate in a glass bottom dish, with 1.0 mL (1.0 × 10^4^ cells) of cell suspension in each dish. After cell adhesion, culture medium was removed. The cell layer was rinsed twice with phosphate buffered saline (PBS) (pH 7.0), and then treated with **ARC** or **ARC** and **DCP** according to the experimental need.

#### Cell imaging study

For confocal imaging studies, 1 × 10^4^ A549 cells in 1000 μL of medium, were seeded on sterile 35 mm glass bottom culture dish (ibidi GmbH, Germany), and incubated at 37 °C in a CO_2_ incubator for 10 hours. Then cells were washed with 500 μL DMEM followed by incubation with **ARC** (1 µM) dissolved in 1000 μL DMEM at 37 °C for 1 h in a CO_2_ incubator and cells were washed thrice with phosphate buffered saline (PBS) (pH 7.0) to remove excess **ARC** observed under an Olympus IX81 microscope equipped with a FV1000 confocal system using 1003 oil immersion Plan Apo (N.A. 1.45) objectives. Images obtained through section scanning were analyzed by fluorochrome filter POPO-3 with excitation at 534 nm monochromatic laser beams, and emission spectra were integrated over the range 580 nm (single channel). The cells were again incubated with **DCP** (10 µM) for 10 min and excess **DCP** was washed thrice with PBS (pH 7.0) followed by observations under microscope. For all images, the confocal microscope settings, such as transmission density, and scan speed, were held constant to compare the relative intensity of intracellular fluorescence. All images were captured using 2% lesser light.

#### Cytotoxicity assay

*In vitro* studies established the ability of the chemosensor **ARC** to detect **DCP** in biological system. Human cell A549 (ATCC No CCL-185) were used as models. However, to materialize this objective, it is a prerequisite to assess the cytotoxic effect of **ARC** and **ARC-DCP** complex on live cells. The well-established MTT assay was adopted to study cytotoxicity of above mentioned complexes at varying concentrations. A cytotoxicity measurement for each experiment shows that the chemosensor **ARC** does not have any toxicity on the tested cells and **ARC-DCP** complex does not exert any significant adverse effect on cell viability at tested concentrations.

### Imaging of Catfish brain

#### Methodology

Live specimens of adult male catfish, *Clarias batrachus* (Actinopterygii, Siluriformes, Clariidae), average body weight 65.48 ± 3.91 g, were maintained in laboratory condition as described earlier^43^. All the experimental protocols were approved by the Institutional Animal Ethical Committee (Reg. no.: 1819/GO/Ere/S/15/CPCSEA) of Visva-Bharati University, Santiniketan, India. We also confirm that all the experiments were performed in accordance with the relevant guidelines and regulations. During autopsy, fishes were killed by decapitation; brain was dissected out and placed immediately in phosphate buffer saline, PBS (0.1 M phosphate buffer; pH 7.4 containing 0.6% NaCl and 0.01% Triton X-100). Catfish brain, specifically the optic tectum (telencephalon) region was sliced using sharp blades and incubated in 6 well culture plate (SPL, India), either in PBS alone or supplemented with the **ARC** (1 µM) for 15 min in a humidified chamber at 23 ± 2^0^ C. Brain slices were washed thrice with PBS, probed with the guest (**DCP**: 10 µM), for 5 min with gentle shaking (40 rpm), washed again to remove unbound guest and observed under an inverted fluorescent microscope (DMi8, Leica Microsystems, Germany) using HI PLAN I 10 × /0.22 PH1 objective and ROHD filter (excitation and emission ranges at 520 nm and 580 nm respectively). Images were acquired through Leica EC4 Digital Camera and DFC 3000 G and processed using LAS X software.

## Electronic supplementary material

Supplementary Information
