# Supplementary material for: Visualisation of DCP, a nerve agent mimic, in Catfish brain by a simple chemosensor
Source: Sci Rep. 2018 Feb 21;8:3402. doi: 10.1038/s41598-018-21780-5 (PMC5821826; doi:10.1038/s41598-018-21780-5)
Supplement: Supplementary file 1 — Supplementary Information [file 41598_2018_21780_MOESM1_ESM.pdf]

Supplementary Information

Visualisation of DCP, a nerve agent mimic, in Catfish brain by a simple chemosensor

Himadri Sekhar Sarkar[1], Ayndrila Ghosh[1], Sujoy Das[1], Pulak Kumar Maiti[2], Sudipta Maitra[3], Sukhendu Mandal[2] & Prithidipa Sahoo[1]

[1]Department of Chemistry, Visva-Bharati University, Santiniketan, 731235, India. [2]Department of Microbiology, University of Calcutta, Kolkata-700019, India. [3]Department of Zoology, Visva-Bharati University, Santiniketan, 731235, India.
Correspondence and requests for materials should be addressed to P.S. (email: prithidipa@hotmail.com)

Table S1. Performance comparison of existing methods and present method for detection of **DCP**.

Analytes	Sensor type	Detection limit	Medium	Detection method	Detection state	*In vivo* experiment	References
**DCP**	**Rhodamine**	**5.6 nM**	**Basic medium**	**Naked-eye, UV-Vis, Fluorescence**	**Liquid/Vapour state**	**Yes**	**Present manuscript**
DCP, DCNP	Triaryl methane dye	2.1-3.2 mmol dm^{-3}	Acidic medium	Naked-eye, UV-Vis	Liquid state	No	*Tetrahedron* **68**, 8612-8616 (2012)
DCP	Rhodamine	0.2 µM	Basic medium	Naked-eye, UV-Vis, Fluorescence	Vapour state	No	*RSC Adv.* **4**, 21984-21988 (2014)
DFP, DCNP	BODIPY	0.36 and 0.40 ppm	-	Naked-eye, UV-Vis	Liquid/Vapour state	No	*Org. Biomol. Chem.* **12**, 8745-8751 (2014)
DCP	Rhodamine-thiourea	0.14 µM	Basic medium	Naked-eye, UV-Vis, Fluorescence	Liquid/Vapour state	No	*Sensors and Actuators B: Chemical* **235**, 447 (2016)
DCP	Napthothiazolium conjugated benzothiazole derivative (NTBT)	17 nM	-	Naked-eye, UV-Vis, Fluorescence	Liquid/Vapour state	No	*Chem. Commun.* **51**, 9729-9732 (2015)
DFP	Organo silyl ether	5.4 ppm	Basic medium	Naked-eye, UV-Vis, Fluorescence	Liquid state	No	*Chemistry Open* **3**, 142 (2014)
DCP	Rhodamine-hydroxamate	ND	Basic medium	Naked-eye, UV-Vis, Fluorescence	Liquid state	No	*Chem. Commun.* **46**, 8413–8415 (2010)
DCP	Rhodamine-deoxylactam	ND	Basic medium	Naked-eye, UV-Vis, Fluorescence	Liquid state	No	*Chem. Commun.* **47** 11468–11470. (2011)
DCP	Hydroxynaphthalene-hemicyanine dye	18.86 nM	Basic medium	Naked-eye, UV-Vis, Fluorescence	Liquid/Vapour state	No	*Org. Biomol. Chem.* **15**, 5959 (2017)

2. NMR Studies

^{1}H NMR of ARC in DMSO-d_6:

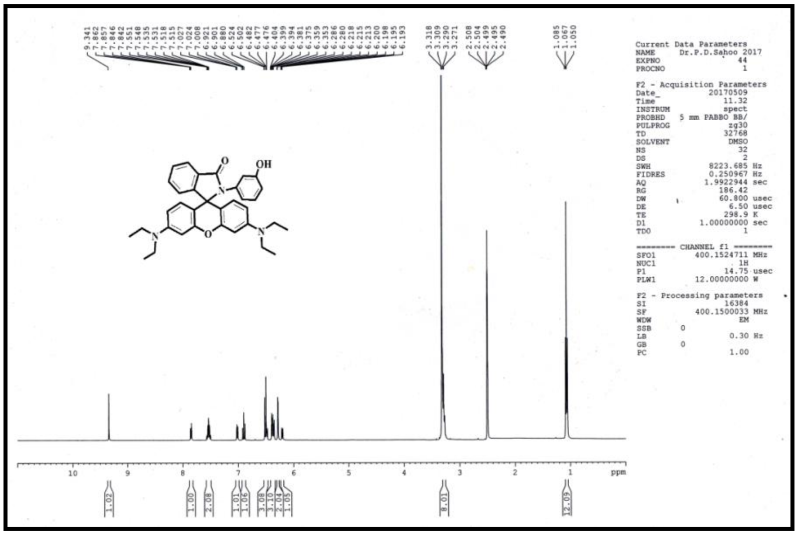

Figure S1. ^{1}H NMR of **ARC** in DMSO-d_6 (400 MHz).

^{13}C NMR of ARC in DMSO-d_6:

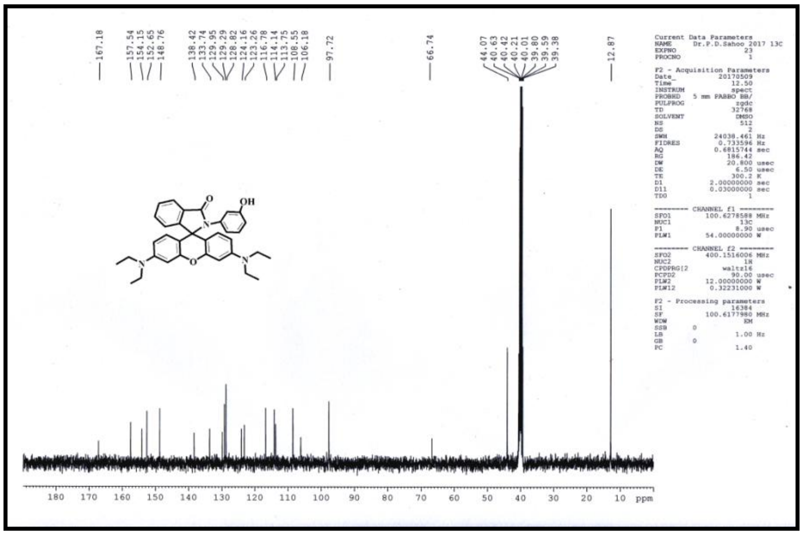

Figure S2. ^{13}C NMR of **ARC** in DMSO-d_6 (400 MHz).

Mass spectrum of ARC:

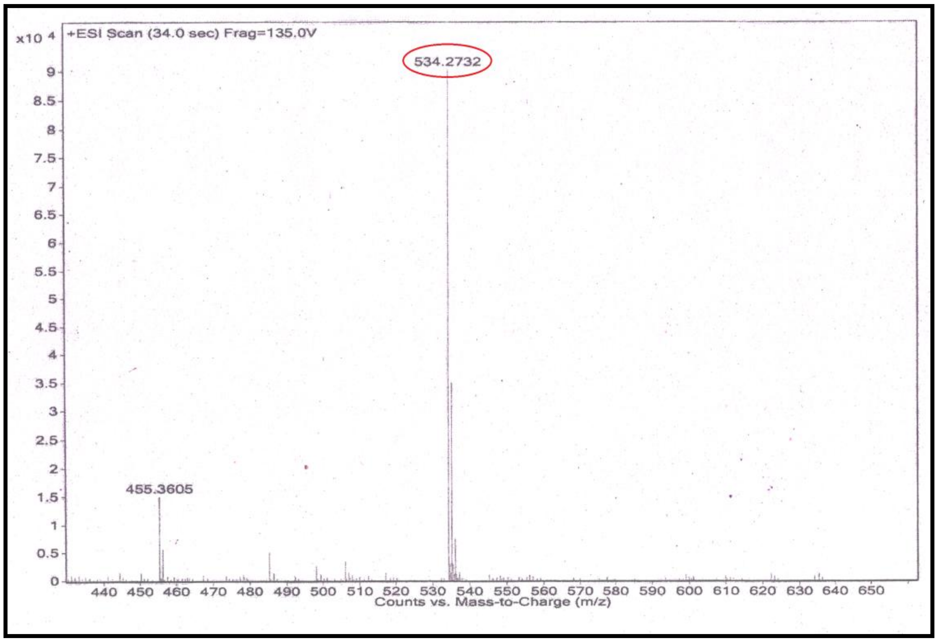

Figure S3. HRMS of **ARC**.

Measurement of fluorescence quantum yields[1]:

The fluorescence quantum yield (QY) of **ARC** was determined relative to a reference compound of known QY. Rhodamine B (Φ_F = 0.69 in ethanol) as a reference compound because it has emission profile between 500-600 nm similar to **ARC**. As shown in **Table S2**, the quantum yield of **ARC** increased upon addition of **DCP**. Almost 100-fold fluorescence intensity increased upon addition of **DCP**.

Table S2. Comparison of quantum yields of **ARC** and **ARC-DCP** complex

**Sample**	**Quantum yield at 585 nm***
**ARC**	0.006 ± 0.001
**ARC-DCP** complex	0.58 ± 0.03

***Average value of three determinations.

Evaluation of the association constants for the formation of ARC-DCP complex:

By Fluorescence Method:

Binding constant of the chemosensor **ARC** was calculated through emission method by using the following equation:

$$1/ (I - I_0) = 1/K(I_{max} - I_0) [G] + 1/(I_{max} - I_0) \quad \ldots\ldots\ldots\ldots\ldots(ii)$$

Where I_0, I_{max}, and I represent the emission intensity of free **ARC**, the maximum emission intensity observed in the presence of added **DCP** at 585 nm (λ_{ex}= 520 nm), [G] is the concentration of the guest **DCP** and the emission intensity at a certain concentration of the **DCP**, respectively. [H] is the concentration of the host **ARC**.

Binding constant calculation graph (Fluorescence method):

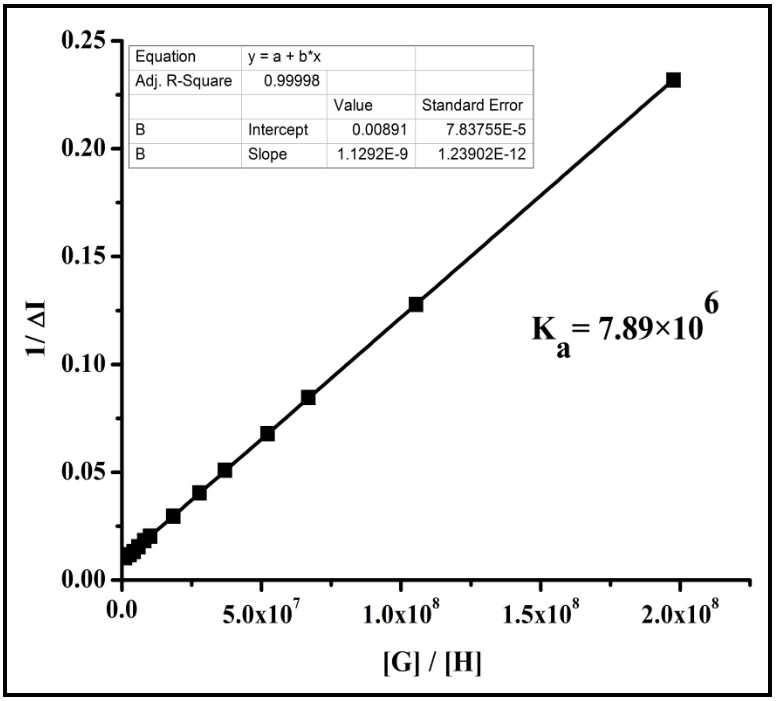

Figure S4. Linear regression analysis (1/[G] vs 1/ΔI) for the calculation of association constant value by fluorescence titration method.

The association const. (K_a) of **ARC** for sensing **DCP** was determined from the equation: K_a = intercept/slope. From the linear fit graph we get intercept = 0.00891, slope = 1.1292×10^{-9}. Thus we get, $K_a = 0.00891 / (1.1292 \times 10^{-9}) = 7.89 \times 10^6$ M^{-1}.

Calculation of limit of detection (LOD) of ARC with DCP:

The detection limit of the chemosensor **ARC** for **DCP** was calculated on the basis of fluorescence titration. To determine the standard deviation for the fluorescence intensity, the emission intensity of four individual receptors without **DCP** was measured by 10 times and the standard deviation of blank measurements was calculated.
The limit of detection (LOD) of **ARC** for sensing **DCP** was determined from the following equation[2-3]:

$$LOD = K \times SD/S$$

Where K = 2 or 3 (we take 3 in this case); SD is the standard deviation of the blank receptor solution; S is the slope of the calibration curve.

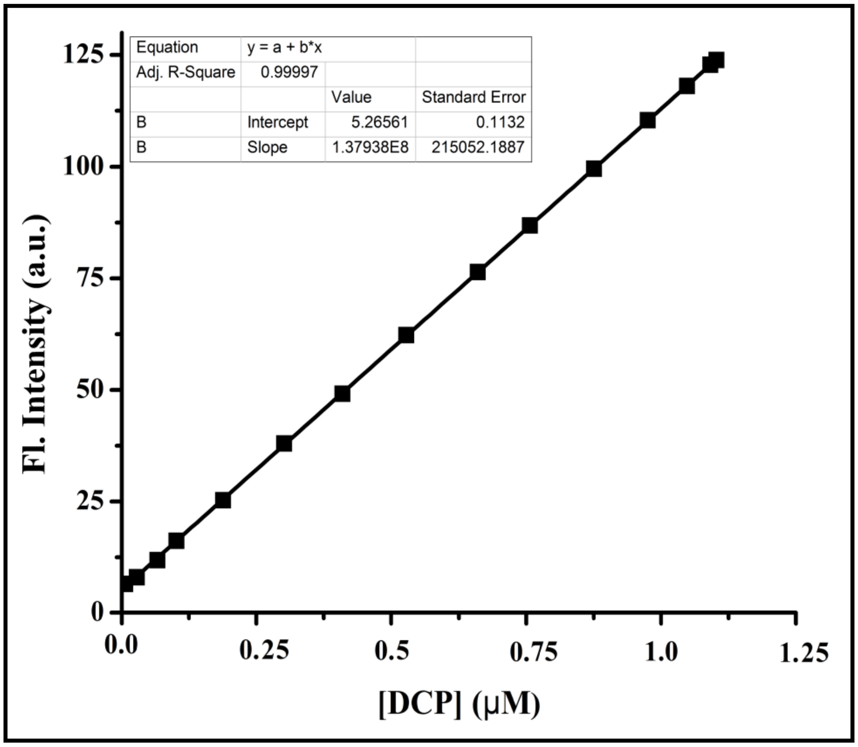

Figure S5. Linear fit curve of **ARC** at 585 nm with respect to **DCP** concentration.

For **ARC** with **DCP:**
From the linear fit graph we get slope = 1.37938×10^8, and SD value is 0.26037.
Thus using the above formula we get the Limit of Detection = 0.56×10^{-8} M, i.e 5.6 nM. Therefore **ARC** can detect **DCP** up to this very lower concentration by fluorescence technique.

Selectivity studies:

Comparative UV-vis studies of ARC with various organophosphates:

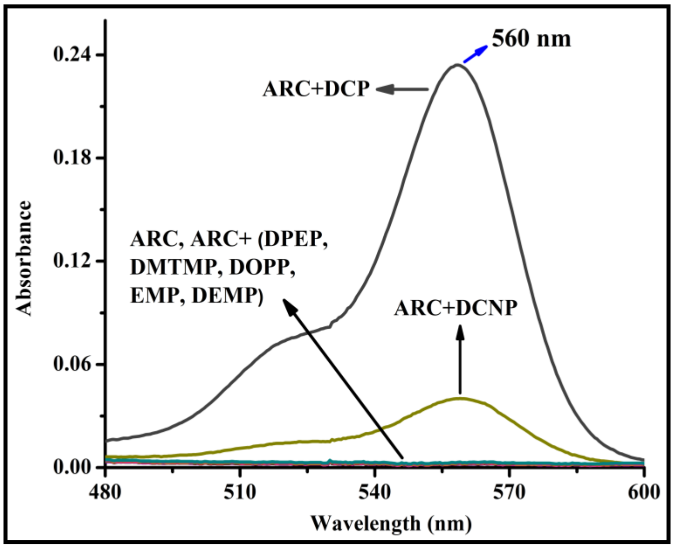

Figure S6. UV-vis spectra of **ARC** (1 μM) upon addition of different organophosphates in H_2O-CH_3CN (10:1, v/v) at neutral pH (Guests conc. = 10 μM). [From left to right: **ARC**, **ARC** with-**DCP**, diethylcyanophosphonate (DCNP), diethyl(1-phenylethyl)phosphonate (DPEP), diethyl(methylthiomethyl)phosphonate (DMTMP), diethyl-(2-oxopropyl)phosphonate (DOPP), Ethyl methylphosphonate (EMP), Diethyl methylphosphonate (DEMP)].

Competitive fluorescence studies of ARC with various organophosphates:

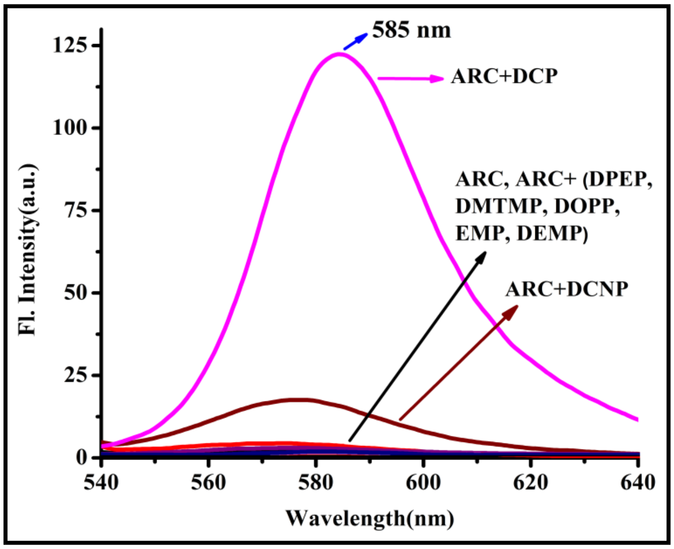

Figure S7. Fluorescence emission spectra of **ARC** (1 μM) upon addition of different organophosphates at 585 nm (λ_{ex}= 520 nm) in H_2O-CH_3CN (10:1, v/v) at neutral pH (Guests conc. = 10 μM).

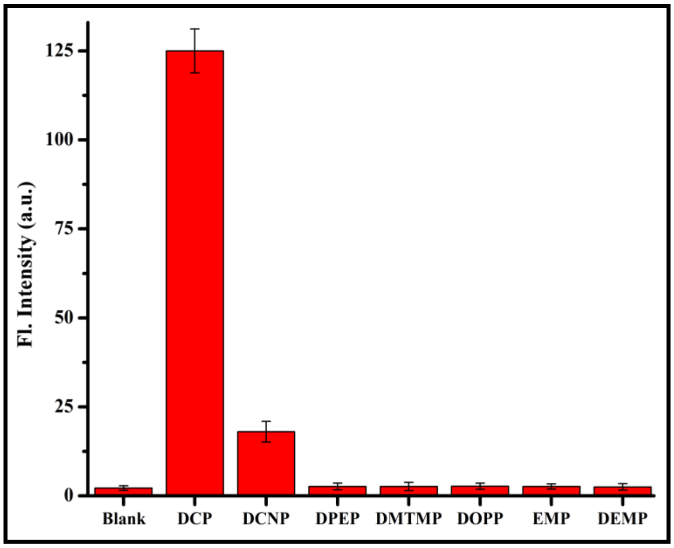

Figure S8. Histogram representing competitive fluorescence spectra of **ARC** with different organophosphates at 585 nm (λ_{ex}= 520 nm) in H_2O-CH_3CN (10:1, v/v) at neutral pH. Error bars represent standard deviations (n = 3).

Competitive fluorescence studies of ARC with various interfering substances and metal ions:

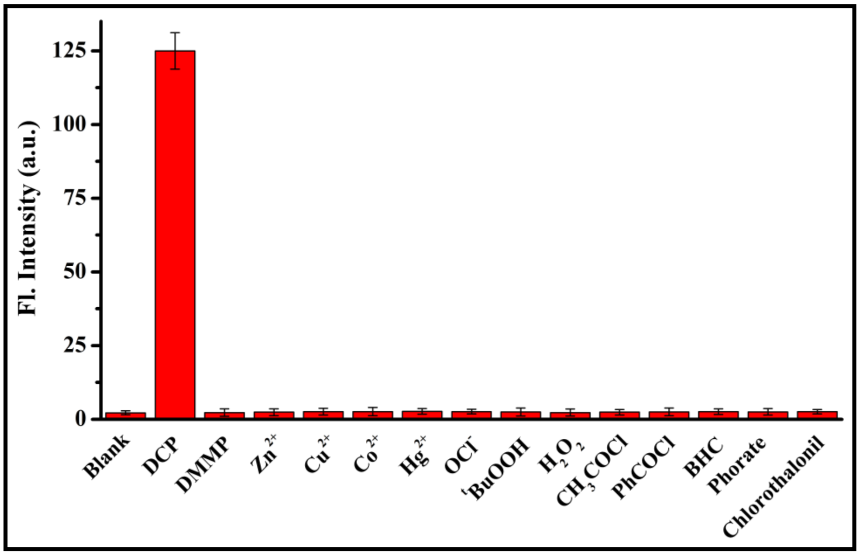

Figure S9. Histogram representing competitive fluorescence spectra of **ARC** with various interfering substances and metal ions at 585 nm (λ_{ex}= 520 nm) in H_2O-CH_3CN (10:1, v/v) at neutral pH. [From left to right: **ARC**, **ARC** with- **DCP**, dimethylmethylphosphate (DMMP), Zn^{2+}, Cu^{2+}, Co^{2+}, Hg^{2+}, NaOCl, ^tBuOOH, H_2O_2, CH_3COCl, PhCOCl, benzene hexachloride (BHC), phorate and chlorothalonil]. Error bars represent standard deviations (n = 3).

Kinetic study:

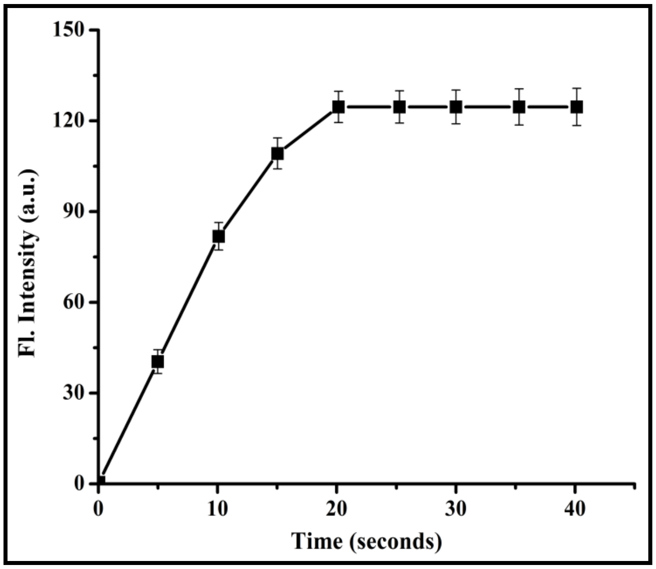

Figure S10. The fluorescence intensities at 585 nm vary in a time range of 40 s. The excitation wavelength was set at 520 nm. Each point of the plot represents the average of at least 3 independent kinetic experiments.

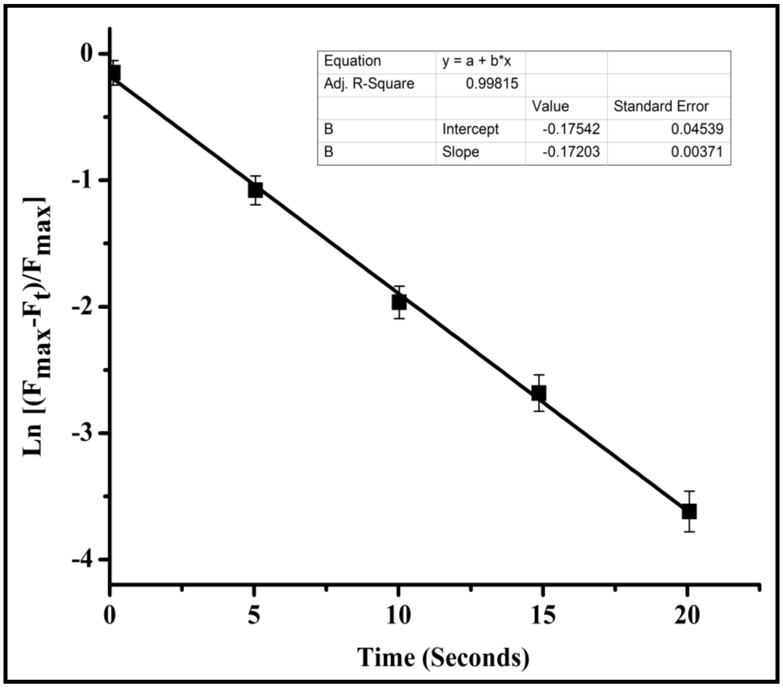

Figure S11. Pseudo first-order kinetic plot for the reaction of **ARC** (1 μM) with **DCP** (10 μM). Each point of the plot represents the average of at least 3 independent kinetic experiments.

Vapour phase detection of DCP by ARC:

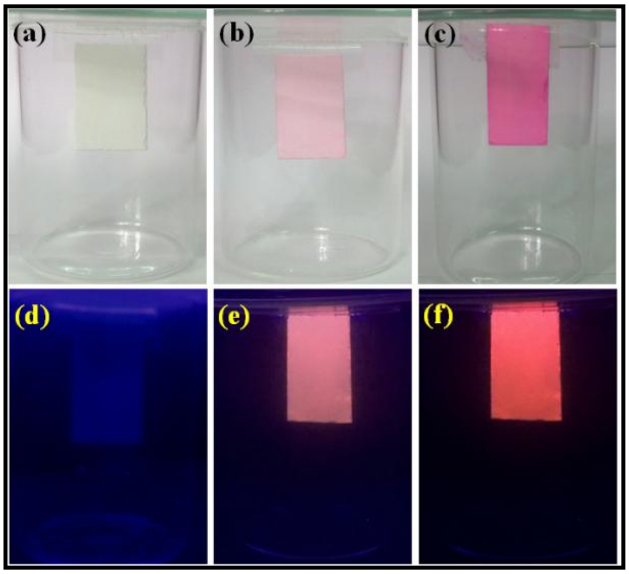

Figure S12. Display of vapour phase sensing of **DCP** using an **ARC** coated paper strip in visual (top) and under a UV lamp (bottom): (a) only an **ARC** coated filter paper; (b) after 15 seconds of incubation of **DCP**; (c) after 30 seconds of incubation of **DCP**; d, e and f are the UV-photographic images of a, b and c respectively.

pH titration study:

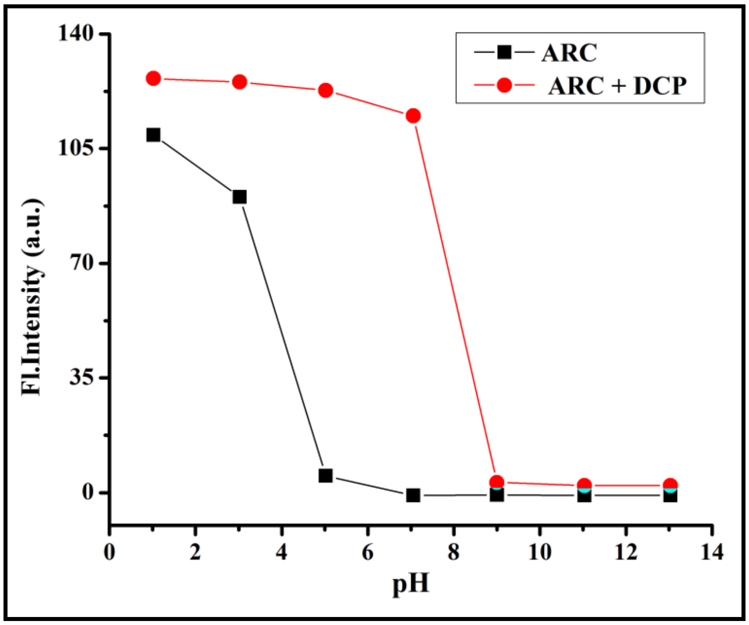

Figure S13. Effect of pH on the fluorescence intensity of **ARC** (1 μM) in the absence of **DCP** (black line) and in the presence of **DCP** (10 μM, red line).

^{1}H NMR titration spectrum of ARC with DCP:

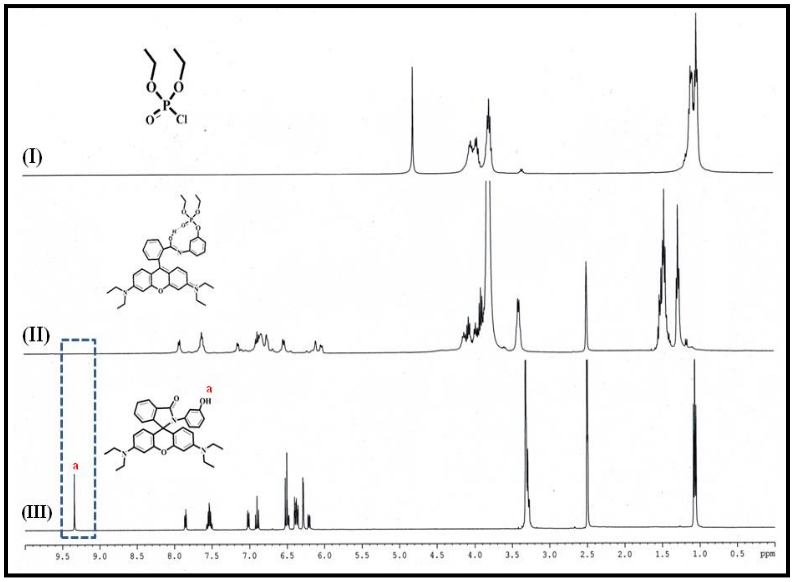

Figure S14. ^{1}H NMR titration [400MHz] of **ARC** in DMSO-d$_6$ at 25^{0}C and the corresponding changes after the addition of 1 equiv. of **DCP** in D_2O from (i) only **DCP**, (ii) **ARC** + 1 equiv. of **DCP**, (iii) only **ARC**.

^{13}C NMR titration spectrum of ARC with DCP:

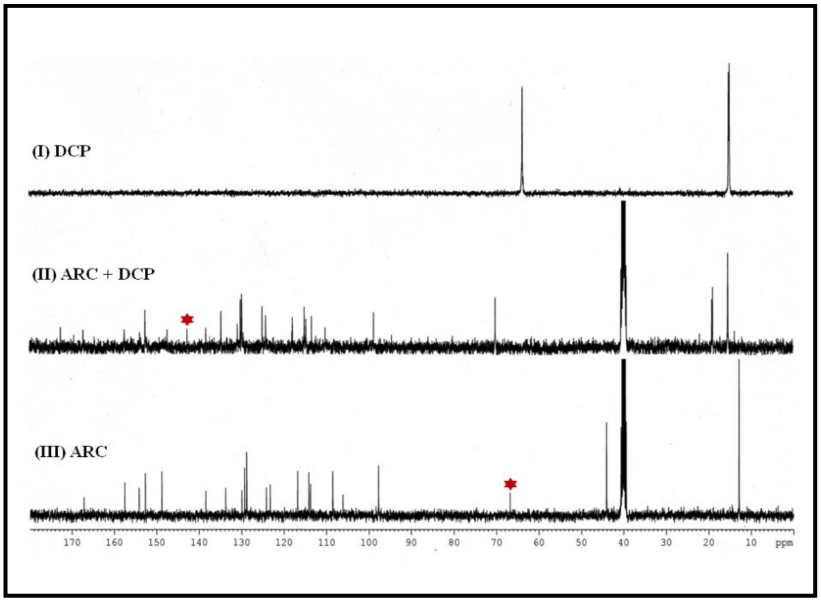

Figure S15. ^{13}C NMR titration [400MHz] of **ARC** in DMSO-d$_6$ at 25^{0}C and the corresponding changes after addition of one equiv. of **DCP** in D_2O from (i) only **DCP**, (ii) **ARC** + 1 equiv. of **DCP**, (iii) only **ARC**. The red spot indicates the shifting of the spiro cyclic carbon peak from 67 ppm to 143 ppm in the open form.

^{31}P NMR titration spectrum of ARC with DCP:

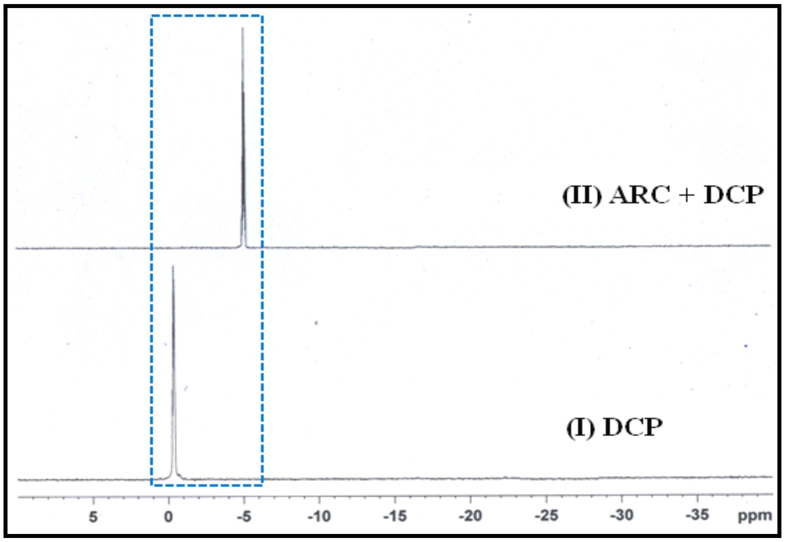

Figure S16. ^{31}P NMR titration [400MHz] of **ARC** in DMSO-d_6 at 25^{0}C and the corresponding changes after addition of one equiv. of **DCP** in D_2O from (I) only **DCP**, (II) **ARC** + 1 equiv. of **DCP**.

DFT Study:

Binding of **ARC** and **DCP** has been investigated by quantum chemical calculations at the TDDFT level 6-31G+(d,p) method basis set implemented at Gaussian 09 program. Solvent effects were incorporated using CPCM solvent model. Geometry optimization resulted in conformational changes at the spiro-lactam position of **ARC**, while **DCP** takes part to accommodate a probe molecule to get the stable complex stucture. This theoretical study strongly correlates the experimental findings.

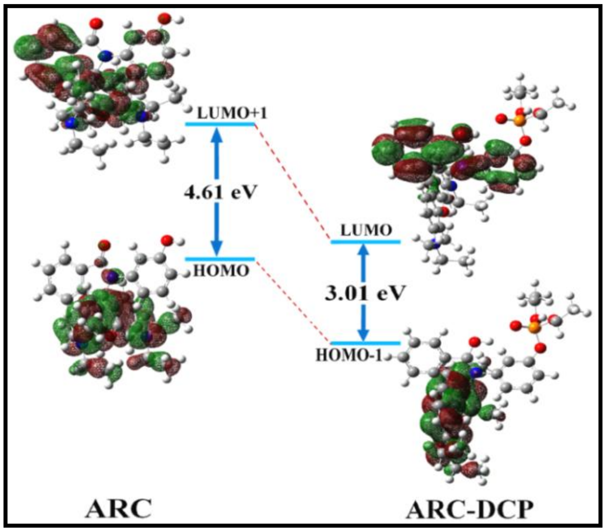

Figure S17. HOMO and LUMO distributions of **ARC** and **ARC-DCP** complex.

Table S3. Selected electronic excitation energies (eV), oscillator strengths (f), main configurations of the low-lying excited states of all the molecules and complexes. The data were calculated by TDDFT//B3LYP/6-31G+(d,p) based on the optimized ground state geometries.

Molecules	Electronic Transition	Excitation Energy[a]	f[b]	Composition[c] (%)
**ARC**	$S_0 \rightarrow S_{12}$	4.0183 eV 308 nm	0.1688	H → L+1 (69.1%)
	$S_0 \rightarrow S_9$	3.6297 eV 341.58 nm	0.0839	H -2 → L (36.8%)
**ARC-DCP**	$S_0 \rightarrow S_1$	2.7695 eV 553.03 nm	0.7625	H -1 → L (73.4%)
	$S_0 \rightarrow S_7$	3.7707 eV 329 nm	0.1048	H → L+1 (49.5%)<br>H -6 → L (35.4%)

[a]Only selected excited states were considered. The numbers in parentheses are the excitation energy in wavelength. [b]Oscillator strength. [c]H stands for HOMO and L stands for LUMO.

Table S4. Energies of the highest occupied molecular orbital (HOMO) and lowest unoccupied molecular orbital (LUMO).

**Species**	**E_{HOMO} (a.u)**	**E_{LUMO} (a.u)**	**ΔE(a.u)**	**ΔE(eV)**	**ΔE(kcal/mol)**
**ARC**	-0.19073	-0.02106	0.16967	4.61	106.46
**ARC-DCP**	-0.29294	-0.18240	0.11054	3.01	69.36

Live Cell Imaging:

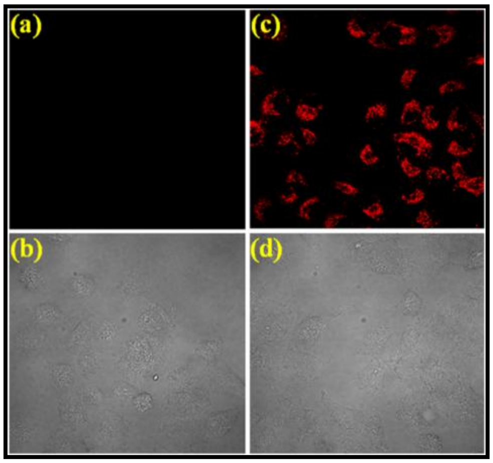

Figure S18. Confocal microscopic images of A549 cells (Human cell A549, ATCC No CCL-185) treated with **ARC** and **DCP** (a) Cells treated with **ARC** at 1 µM concentration. (b) Bright field image of (a). (c) Cells treated with **ARC** and **DCP** at concentration 10 µM. (d) Bright field image of (c). All images were acquired with a 60x objective lens with the applied wavelengths: E_{ex}= 534 nm, E_{em}= 572 nm. Filter used: POPO-3.

Cytotoxicity Assay:

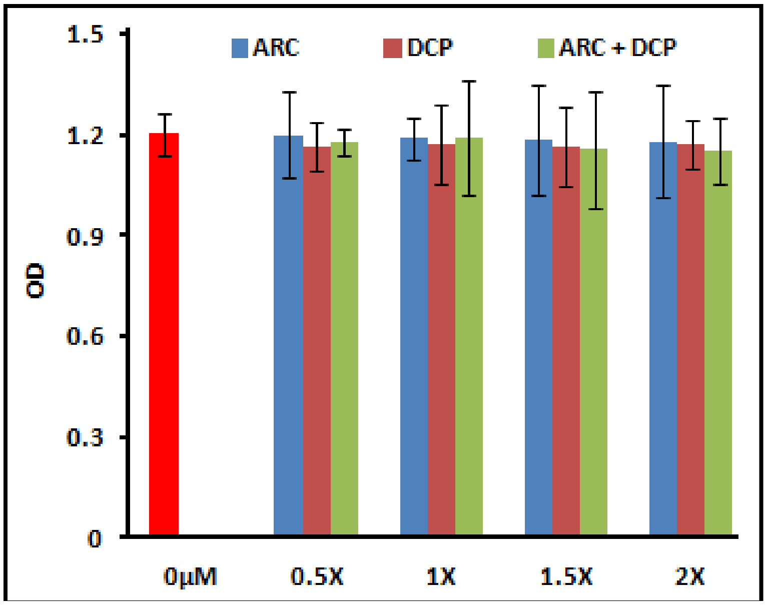

Figure S19. MTT assay to determine the cytotoxic effect of **ARC**, **DCP** and **ARC-DCP** complex on A549 cells (Human cell A549, ATCC No CCL-185).

References:

1. Williams, A. T. R., Winfield, S. A. & Miller, J. N. Relative fluorescence quantum yields using a computer-controlled luminescence spectrometer. *Analyst* **108**, 1067-1071 (1983).
2. Long, L. *et al.* A fluorescence ratiometric sensor for hypochlorite based on a novel dual-fluorophore response approach. *Anal. Chim. Acta.* **775**, 100 (2013).
3. Zhu, M. *et al*. Visible Near-Infrared Chemosensor for Mercury Ion. *Org. Lett.* **10**, 1481 (2008).
4. Nath, P. & Maitra, S. Role of two plasma vitellogenins from Indian major carp (Cirrhinus mrigala) in catfish (Clarias batrachus) vitellogenesis. *General and Comparative Endocrinology* **124**, 30-44 (2001).
